# Congenital upper limb difference patient registries: characteristics, comparisons and recommendations

**DOI:** 10.1177/17531934251348360

**Published:** 2025-06-12

**Authors:** David McCombe, Lindley Wall, Charles Goldfarb, Wiebke Hülsemann, Ida Neergård Sletten, Daniel Wilks, Maxim D Horwitz, Wee Leon Lam

**Affiliations:** 1 Australian Hand Difference Register, Murdoch Children’s Research Institute, Australia; 2 Department of Orthopaedic Surgery at Washington University School of Medicine, USA; 3 Hand Surgery Department, Children’s Hospital Wilhelmstift, Germany; 4 Division of Orthopaedic Surgery, Oslo University Hospital, Norway; 5 United Kingdom Hand Registry, Chelsea and Westminster Hospital, UK; 6 Department of Plastic and Reconstructive Surgery, Royal Hospital for Children and Young People, UK

**Keywords:** Congenital, limb difference, patient registry, patient rated outcomes

## Abstract

Clinical registries that allow longitudinal patient follow-up with standardized outcome measures are useful tools for collecting data that can be used to inform patients and clinicians about the aetiology, natural history and response of various conditions to treatment. Registries are being employed across the world for children with congenital upper limb differences where the benefits of accumulated data for this heterogenous group of significant conditions are proving invaluable including the Congenital Upper Limb Difference registry in the United States, the Congenital Upper Limb Anomaly North registry in northern Europe, the Australian Hand Difference Register in Australia and the British Society for Surgery of the Hand Registry in the UK. These registries collect similar data allowing effective interoperability while retaining individual features unique to each registry. Recommendations for further development are made based on analysis of the development and methodology of these existing registries.

## Introduction

A clinical registry is a database comprising predetermined data collected either from or about individuals who share a defined feature such as a clinical diagnosis or specific treatment or procedure. As technology has advanced, data collection and collation have become more comprehensive, reliable and accessible. The power of clinical registries to provide a more holistic perspective of the impact of a condition has been recognised (Gilklich et al., 2014) Successful registries share several important features: rigorous data management; broad participation from stakeholders; systems to ensure accurate data entry; robust data protection; and, in those registries that focus on longitudinal data collection, maintenance of follow-up at the prescribed intervals. It is also important to maintain appropriate accessibility to the data. The key features of data registry design and management have been codified by Wilkinson et al. in the FAIR (findable, accessible, interoperable and resuable) guiding principles for data management (Wilkinson et al., 2016).

Congenital upper limb differences (CULDs) are a heterogenous group of conditions with various aetiologies, presentations and associations. The numbers of such conditions vary according to countries and even different institutions within a country. As such, a CULD registry would be useful in collating data for analysis. Over the last decade or so, several independent clinical registries of CULD patients have been developed in the various regions of the world. When large datasets are collected, demographic data can help identify specific risk factors for the various CULDs as well as elucidating their respective aetiologies. The registries also prospectively collect outcome data, including patient-rated outcome questionnaire data, which can inform clinicians and patients in their choices of treatment and the impact that their hand difference is likely to have in the long term.

While the design of a particular registry will vary according to the local resources available and its regulatory environment, importantly the current CULD registries share enough features to allow effective comparison of data and provide an international perspective of the impact of CULDs.

This article describes the development and methodology employed by four large multicentre registries of CULD patients to illustrate the potential for further international collaboration.

## Method

The multicentre registries of CULD patients collecting demographic, diagnostic, treatment and outcome data currently are the Congenital Upper Limb Difference (CoULD) registry based in the United States (Bae et al., 2018), the Congenital Upper Limb Anomaly (CULA) North registry in the Scandinavian countries, Finland and Germany (Sletten et al., 2022), the British Society for Surgery of the Hand (BSSH) UK Hand registry and the Australian Hand Difference Register (AHDR) (O’Keefe et al., 2022; Wilks and McCombe, 2024).

There are a number of other registries publishing their results, but these are usually either based in single institutions or are regional or national birth defect registries that record diagnosis without facility for collection of follow-up data. A collaboration of national CULD registries, similar to the CULA North registry, is evolving in the Asia-Pacific region but this has not yet been formalized.

For the purpose of this article, a representative of each of the four multicentre registries noted above was emailed a series of questions (Table 1). The collated answers are presented below and summarised in Table 2.

**Table 1.** Questions asked of the representatives of each of the CULD registries

	Questions
1	What is the history of your registry’s development?
2	What is the purpose of your registry?
3	What is the method of recruitment of patients? Do you have inclusion and exclusion criteria?
4	What classification system is used to organise patients?
5	What data is collected from the patient? Do you obtain data by linking to other data sources such as the health record or other databases?
6	Do you collect outcome measurements or patient rated outcome questionnaire data? If so, when?
7	Do you have an established method of data audit or cleaning?
8	What funding sources do you have and what is your registry infrastructure?
9	How do you manage your research output? What is the mechanism for data sharing or accessing the registry data?
10	What are your perspectives on the strengths and weaknesses of your registry?

**Table 2.** Summary of the characteristics of each of the CULD registries

	CoULD	AHDR	CULA North	BSSH
Established	2014	2017	2018	2019
Recruitment	Opt in recruitment offered to all congenital upper extremity difference patientsType B Ulnar polydactyly followed for 1 year only, otherwise followed to 18 yearsDiagnostic and basic identification data retained for patients who elect not to participate	Opt in recruitment offered to all congenital upper extremity difference patients.Diagnostic and basic identification data retained for patients who elect not to participate	Opt in recruitment offered to all congenital upper extremity difference patientsDiagnostic and basic identification data retained for patients who elect not to participate	Opt in recruitment offered to all congenital upper extremity difference patients.
Exclusion criteria	Previous surgical treatment of the affected limb	Non-English speakers unable to access translation services	Not fluent in national language if translators not available	
Classification	OMTSubclassifications for specific diagnoses	OMT	OMTICD-10Subclassifications for specific diagnoses	OMT
Patient Data collected	See Table 3	See Table 3	See Table 3	ICHOM CULA dataset
Outcome Data collected	*(a) Patient-rated outcome data*PROMIS® Upper extremity function; Pain; Depression, Anxiety and Peer Relations questionnaires every 3 years from age 5 to 17 yearsPODCI every 3 years from age 3 to 17 years*(b) Surgery performed(c) Complications*	*Patient-rated Outcome data*PROMIS®Upper extremity function; Pain; Depression, Anxiety, Global Health and Peer Relations questionnairesICHOM CULA data set appearance questionnaire at 5,8, 11,14 and 17 years of age	Data collected at 1,2 , 6, 8, 12 and 15 years of age and day before all surgeriesData collected includes:*(a) age-stratified physical assessment of limb motion,* grasp pattern and strength*(b) Patient-rated outcomes*PROMIS® Upper extremity function; Global Health; Depression, Anxiety and Peer Relations questionnaires at 6,8,12 and 15 yearsAppearance questions at age 1, 2, 6, 8, 12 and 15 yearsPain scale at 12 and 15 years*(c) Burden of care*Number of clinic visitsNumber of orthoses fittedDetails of surgeries performed	ICHOM CULA data setData collected at 6 months,1,2,6,8,12, 15 years and end of paediatric care**a. Treatmentb. Burden of care**Major complicationsMedical admissions**c. Degree of Health**Upper Limb function: goniometry, manual muscle testing, dynamometryPatient Rated Outcomes:PROMIS® Upper extremity function; Global Health; Depression, Anxiety and Peer Relations questionnaires at 6,8,12 and 15 years and end of careAppearance questions at age 6,8,12, 15 years and end of care

CoULD, Congenital Upper Limb Difference, registry based in the United States; CULA, Congenital Upper Limb Anomaly North registry in the Scandinavian countries, Finland and Germany; AHDR, Australian Hand Difference Register; BSSH, British Society for Surgery of the Hand, UK; OMT: Oberg, Manske and Tonkin classification

## Results

### History of registry development

The four CULD multicentre registries have been established over the last 11 years with the CoULD registry beginning recruitment in 2014, the AHDR in 2017, the CULA North registry in 2018 and the BSSH UK Hand Registry in 2019. The development of each registry has been distinct, reflecting the unique environments within which they operate. The CoULD, CULA North and AHDR registries were developed as standalone congenital registries with independent administration and have grown incrementally through the addition of further recruitment centres. For the CoULD registry, recruiting centres apply to participate and provide evidence of clinical volume and having adequate research support to allow for recruitment and data collection. These centres then undergo an onboarding process supervised by a committee within the CoULD structure. These steps allow for uniform and robust data collection. The CULA North registry operates as a collaboration of several national registries pooling data. The AHDR was developed as a collaboration between the clinicians at the Royal Children’s Hospital and the researchers of the Murdoch Children’s Research Institute, piloted through the Royal Children’s Hospital, and then rolled out to the other dedicated paediatric hand surgery units in Australia. The BSSH congenital hand registry was developed as a subset of the comprehensive UK Hand Registry administered by the BSSH.

### Purpose of the registry

Establishing the purpose of a registry is an important step for its design. There are subtle differences between the registries as they have been developed independently. The stated goals of the CoULD registry are ‘to describe the epidemiology, clinical characteristics, function and health status of children with congenital upper limb differences and the quantification of improvements with non-operative and surgical care’ (Bae et al., 2018). The goals of both the AHDR and the CULA North are to collect epidemiological data and recruit cohorts of patients with specific anomalies or who had undergone specific surgical interventions, and to collect a standardised data set for follow-up of operative and non-operative patients. The AHDR was designed to be a national database to capture population data, and hence, the registry includes all major congenital hand clinics in the country that then refer patients to a centralised database for registration and data collection. The congenital component of the BSSH registry has a different structure as it is part of a broader hand surgery registry that analyses outcomes of treatment of various hand conditions. The BSSH registry is structured to allow individual surgeons to use validated measures to compare their own results with the overall outcomes in the registry.

### Recruitment

The mode of recruitment of patients to the registries varies between registries. Both the CULA North and AHDR registries offer enrolment to patients regardless of whether they have had previous surgery, while the CoULD registry enrols only those patients who have not undergone surgical treatment on at least one upper limb. Enrolment is performed by treating physicians in the CoULD and CULA North registries and by both physicians and therapists in the BSSH and AHDR registries. The AHDR registry also accepts self-referrals from patients with the diagnosis subsequently confirmed by the steering committee based on submitted photographs. Enrolment is an opt-in process for families for all registries. The CoULD, AHDR and CULA North registry have the capacity to retain diagnostic and identifying data (initials, year and state of birth for the CoULD and AHDR registries, and name and identification number for CULA North registry), without requiring patient consent, with a view to establishing population incidence of the various CULDs. The exclusion criterion for recruitment for the CoULD registry is if the child has already undergone surgical treatment for the hand difference(s) prior to enrolment. For the other registries, inability to comprehend the national language of the registry or access translation services are exclusion criteria given the questionnaires are presently only available in the national language of the register.

### Classification

All registries use the updated version of the Oberg–Manske–Tonkin (OMT) classification system (Goldfarb et al., 2020) and include the facility to record multiple diagnoses for the same patient. In addition, the CoULD and CULA North registries further classify children with specific diagnoses, such as radial longitudinal deficiency, using established classification systems. The CULA North registry additionally classifies patients according to the International Classification of Diseases, 10th Revision (World Health Organization, 2004).

### Patient data collected

At recruitment, all registries collect a similar range of data including diagnosis, associated conditions, pregnancy and delivery information, family history including detailed maternal and paternal history, and demographic data about the socio-economic environment of the family (Table 3). The CoULD and CULA North registries include clinical photographs and radiographs of the affected limb(s). The AHDR registry data are gathered by online questionnaire with the option of uploading photographs. The AHDR registry gains consent to obtain access to the patients’ medical record that includes radiographs and clinical photographs. The BSSH registry aims to collect the International Consortium Health Outcome Measurement (ICHOM) CULA dataset which includes clinical examination data as well as questionnaire data, (ICHOM, 2018) and has the facility for establishing links to hospital health records to populate the registry database fields.

**Table 3.** Data gathered from CULD registry participants at enrolment

Data	Information
Family and demographic data	Name
	Address
	Date of birth
	Contact information
	Parental employment and education
	Parental ethnic background
	Parental consanguinity
	Family history of limb difference
Antenatal data	Maternal weight and height
	Pre-existing maternal health conditions
	Number of previous pregnancies and births
	Assisted conception of pregnancy
	Antenatal testing
	Antenatal health
	Antenatal medications/supplements
	Antenatal smoking, alcohol and recreational drug or chemical exposure
Birth data	Gestational age
	Parental age at time of birth
	Type of birth
	Birth weight and length
Clinical photographs and radiographs	N/A
Concurrent conditions	Depends on condition
Treatment	Depends on condition

Following recruitment, all registries collect patient rated outcome (PRO) questionnaire data at prescribed time points. The CoULD registry collects both Patient-Rated Outcome Measurement Information System® (PROMIS®) (Cella et al., 2010) and Pediatric Outcomes Data Collection Instrument (PODCI) (Daltroy et al., 1998) questionnaires at time of study recruitment and then subsequently at 3 yearly intervals. The other registries collect the PROMIS® questionnaires at similar intervals. The individual banks of questions of the PROMIS® questionnaires collected are either the parent-proxy or patient short-form version 2.0 questionnaires including upper extremity function, peer relations, pain interference, depressive symptoms, anxiety and the version 1.0 Global Health questionnaire. In addition to these validated PRO questionnaires, the CULA North, BSSH and AHDR collect the appearance outcomes data using either part of or the whole of the questionnaire proposed in the ICHOM’s Congenital Upper Limb minimum dataset. (ICHOM, 2018). The CULA North registry collects physical assessment data for patients with particular anomalies, including passive range of motion of the elbow, wrist, forearm and hand in children between 1 and 5 years of age, active range of motion in children older than 5 years, forearm length in all children, and the wrist position and stability of the thumb in children with radial longitudinal deficiency. This registry also collects functional assessments of grip and pinch function, pain scores and burden of care data based on hospital visits and treatment. The BSSH registry collects similar data using the ICHOM’s minimum dataset as a guideline.

### Data management and access

Data are maintained on centralised, password protected servers with restricted access by authorised personnel only. The CoULD and AHDR registries use the Research Electronic Data Capture (REDCap) (Vanderbilt University, Nashville, TN, USA) software to manage the data (Harris et al., 2009). Data audit or cleaning is performed intermittently at the CoULD sites but is not performed systematically in any of the other registries. It is performed as part of the investigative process for specific research projects.

The AHDR releases deidentified summary data as part of a trimonthly newsletter circulated to the recruiting centres and patients. The CoULD registry also circulates a summary of registry data in a regular newsletter. The BSSH registry allows individual surgeons to access their own data as an individual audit function. The process of gaining access to registry data for research is similar across registries and requires application to the registry research committee and, depending on the data required, usually approval from an institutional research ethics committee or review board for individual research projects. The AHDR consent for registry enrolment includes consent to access the medical record and contact the patient for future research, allowing for the further investigation of patient cohorts beyond the data collected in the registry.

### Infrastructure and funding

While the use of web-based platforms for data retention limits the infrastructure required to maintain a multicentre registry, personnel are required both to develop the registry framework and for ongoing engagement with the families to facilitate data collection, in addition to data retrieval and analysis. While clinicians may be motivated to commit time to registry development and data analysis, it is often unrealistic to expect this in the face of busy schedules. Clinicians alone are unlikely to be able to adequately manage a significant registry in the longer term. The registries studied have varied personnel infrastructure from centralised administrations (the BSSH and AHDR) to recruiting centre-based research personnel, co-ordinated centrally (CoULD and CULA North). The administrative structure of the registries also varies. The CoULD registry has an executive committee and a subcommittee structure that includes committees for classification, onboarding, data cleaning, international development, website, and research. The CULA North registry has individual administrative committees for each site that liase with the local data protection officers and ethics committees that produce data for studies that are then collated by representatives of each site. The AHDR has a central steering committee that is responsible for recruiting centre engagement and governing registry access. In addition to the data manager/research assistant role in developing and maintaining the registries, there is a need for specialist information technology, statistician and bioethicist support that is usually provided by the supporting research institutions affiliated with the registry. The funding for these registries is derived from institutional budgets in the case of the CoULD registry and CULA North, philanthropy (AHDR) or the sponsoring professional society (BSSH).

## Discussion and recommendations

Clinical registries, particularly multicentre registries as described in this article, are useful research tools that may provide answers to important questions regarding the aetiology and natural history of disease, the effectiveness of treatment(s), and the burden of the disease upon not only the patient but also the community within which the patient lives (Gilklich et al., 2014). For children and families with CULDs, registries offer specific benefit by aggregating knowledge about these rare conditions and reporting treatment outcomes of large groups of affected children. Often, the available information to date has been based on reviews of relatively small cohorts of patients within a single surgeon’s or institution’s practices. The power of these registries is that they can accumulate data to strengthen the value of the conclusions drawn and provide more comprehensive knowledge. For this to occur and for the registries to be effective, they must satisfy several criteria. Ideally a CULD registry is inclusive to avoid the peril of selection bias in its output, sustainable so as to capture long-term outcomes, interoperable with collection of similar domains of data so that comparisons with other registries are feasible, and secure and accessible with appropriate governance and security infrastructure that maintain data integrity and privacy but allow for comprehensive retrieval and reporting of data. The registries surveyed in this study vary in structure and the information they gather according to their local conditions and their goals but share basic features of recruitment, classification, registration data and similar schemes for follow-up and the use of validated PRO measures. In this way these registries satisfy the criteria noted above.

Given the variation in structure and background, the relative strengths and weaknesses vary between registries. The use of an additional PRO, the PODCI, in the CoULD registry enables early outcome collection and prospective comparison between these instruments across their patient population (Wall et al., 2020). The CULA North and BSSH follow-up schedules are more comprehensive than the CoULD and AHDR registries as they collect data in line with ICHOM’s CULA dataset regime including objective as well as subjective data. This reflects the nature of these systems that centralizes care and allows for regular direct review of the patients, whereas in the US and Australia, patient care may be more decentralized and constrained in time available for face-to-face contact, and hence a follow-up schedule that can be employed by online questionnaire requiring less direct follow-up is appropriate.

The challenges of registries in achieving comprehensive recruitment and data collection are similar. The AHDR was established with a goal of achieving a national database to enable both assessment of outcome with regional variation as well as prevalence. This approach was enabled by the regulatory environment that allows for an institutional Human Research Ethics Committee/Institutional Review Board approval to be employed as the basis for approvals for data collection and transfer between institutions. While this structure of a centralized web-based data collection and ongoing follow-up via REDCap notifications to registry participants would seem ideal in minimising the burden for clinicians and individual institutions, it is dependent on clinician and patient (parent) motivation to recruit and then complete follow-up questionnaires. There have been significant numbers of patients lost to follow-up during the outcome data-collection schedule in the AHDR cohort, particularly from the lower socio-economic quintile. The CoULD and CULA North registries have grown incrementally, adding sites that are able to provide adequate research personnel support to maximise patient recruitment and retention. This approach strengthens data capture but limits the reach of these registries to those centres involved. Ultimately this may be a better approach, as the alternative AHDR strategy of trying to achieve nationwide data capture from its inception has led to bias in recruitment to the better resourced centres, skewing the incidence data. The BSSH registry has perhaps the most ambitious schedule for data capture as it uses the full ICHOM CULA dataset; however, this places a significant burden on the clinician and to date has not been widely adopted amongst congenital hand surgeons owing to a shortage of administrative time.

The adage about the quality of research output relating to the quality of data input holds true. The use of the OMT classification system and the PROMIS® outcome measure across these registries is a strength allowing collaboration and comparison between them; however, the OMT classification is not perfect with some patients difficult to classify (Lam et al., 2025; Sait et al., 2022; Sletten et al., 2022). Wall et al. (2024) used a consensus decision-making process to resolve a cohort of unclassifiable patients with 93% success, pointing to the role within each registry of a committee to deal with the difficult to classify cases to improve accuracy and consistency of classification, such as is employed in the CoULD registry. Further tools to improve consistency of diagnosis between registries include the OMT App and the OMT video guide resources which can be found on the International Federation of Societies for Surgery of the Hand (IFSSH) website (https://www.ifssh.info/OMT-Classification-App.php) (Lam, 2025). An international Delphi consensus study is also underway to standardise some of the common disagreements among surgeons when classifying conditions.

Multicentre prospective patient registries for CULD patients are an emerging resource for clinical research and education. It is critical that these registries are designed carefully to satisfy the goals of maximizing patient recruitment and collection of relevant and useful data, particularly long-term outcome data, as we look to collaborate between registries to learn more about the aetiology, treatment and biopsychosocial outcomes for the children with CULD’s and their families.

To allow this interoperability, it is recommended that registries share the following features:

- patient recruitment and classification by experienced clinicians who are able to review either the patient or clinical photographs, together with radiographs where indicated;
- use of the Oberg-Manske-Tonkin system as its predominant classification;
- an expert classification committee to resolve difficult or unclassifiable cases;
- collection of a minimum set of demographic, family history, antenatal and treatment data (Table 2);
- collection of validated patient rated outcome data including PROMIS^®^ questionnaires;
- secure retention of data in a form and facility that protects patient privacy and allows interrogation of the database for specific research queries; and
- a steering or governing committee to assess the merit of research applications, grant authorisation to access registry data, and regularly review the research output of the register

This review describes the characteristics of the current multicentre CULD registries and their strengths and weaknesses so that they can be strengthened and those registries that are under development can do so with the benefit of the lessons learned to date. Ideally, these registries operating across the world will have the capacity to collaborate in CULD research, operating in parallel to produce effectively an international CULD registry.
